# Fundus first as the standard technique for laparoscopic cholecystectomy

**DOI:** 10.1038/s41598-019-55401-6

**Published:** 2019-12-10

**Authors:** Yucel Cengiz, Meisam Lund, Arthur Jänes, Lars Lundell, Gabriel Sandblom, Leif Israelsson

**Affiliations:** 1 Department of Surgical and Perioperative Sciences, Umeå University, SE-851 85 Umeå, Sweden; 2 CLINTEC, Department of Surgery, Karolinska Institutet, SE-171 77 Stockholm, Sweden; 3 Department of Clinical Science and Education Södersjukhuset, Karolinska Institutet, Stockholm, Department of Surgery, Södersjukhuset, SE-118 83 Stockholm, Sweden; 4 Department of Surgery, Odense University Hospital, J.B. Winsloews Vej 4, 5000 Odense, Denmark

**Keywords:** Cholelithiasis, Surgery

## Abstract

In previous studies the fundus first technique (FF) has been a cost-effective way to simplify the laparoscopic cholecystectomy (LC) and facilitate patient rehabilitation. The feasibility and safety profile when introducing FF as the standard technique were aimed in this study. Between 2004–2014, 29 surgeons performed 1425 LC with FF and 320 with a conventional technique. During the first year 56% were with FF and 98% during the last four years. More females, ultrasonic shears, urgent operations, daycare operations and a shorter operation time were found with FF. 63 (3.6%) complications occurred: 10 (0.6%) bleedings, 33 (1.9%) infections and 12 (0.7%) bile leakages. Leakage from cystic duct occurred in 4/112 (3.6%) when closed with ultrasonic shears and in 4/1633 (0.2%) with clips (p 0.008). A common bile duct lesion occurred in 1/1425 (0.07%) with FF and in 3/320 (0.9%) with the conventional approach (p 0.003). In a multivariate regression model, the conventional technique was a risk factor for bile duct injury with an odds ratio of 20.8 (95% CI 1.6–259.2). In conclusion FF was effectively established as the standard procedure and associated with lower rates of bile duct injuries. Clipless closure of the cystic duct increased the rate of leakage.

## Introduction

More than 30 years have passed since the introduction of laparoscopic cholecystectomy (LC). Since then, exponential growths of knowledge, skill and technology have completely changed the clinical practice of gallstone surgery. LC is now the gold standard technique for gallbladder disease in both emergent and elective surgery. Nevertheless, there is neither a wide consensus on its indications nor on its related morbidity and the factors determining associated complications^1,2^.

More than a decade ago^3^, a consensus statement on LC was published. Several main questions were addressed regarding the available evidence and clinical relevance of issues how LC shall be pursued in clinical practice. During the following years, several additional guidelines on LC have emerged including also emergent surgery and concomitant bile duct interventions^4–8^. Noteworthy is that none of these addressed aspects on making the laparoscopic procedure less technically demanding and safer. With the FF technique, the dissection is started at the dome of the fundus and from there the cystic artery and cystic duct are approached. This may sometimes be the technique chosen for LC considered difficult or potentially hazardous due to severe inflammation and/or fibrosis at the triangle of Calot, presence of fatty tissue and portal hypertension^3–13^.

The aim of this study was to evaluate if the FF technique is feasible and if it improves the safety profile. Previous studies have shown that the FF technique is cost-effective, simplifies the procedure and facilitates patient rehabilitation^4,6,14–17^, and a possible improved safety profile has been suggested^13,16,18–20^. With this background the FF technique was launched as the routine technique for all LC at the department. When introducing a new technique, a variety of obstacles may be met, e.g. inexplicable conversion rates, unexpected complications and lack of general acceptance among the staff^13,21^. To evaluate if the FF technique also improves the safety profile all LC during the decade following the introduction of FF as the standard surgical procedure were analyzed.

## Results

From 2004 to 2014, a LC was initiated in 1833 patients at the department. Out of these 43 were very early converted into open surgery as this was regarded safer due to adhesions or other intra operative factors and were excluded from further analysis. Out of those operated on with a conventional technique 4 (1.2%) were converted into open surgery and with the FF technique 30 (2.1%) were converted (p 0.463), due to intra operative factors such as complex anatomy, choledocholithiasis etc.

Patients operated on with the FF technique were more often female, surgery was more often emergent, elective surgery was more often in daycare, dissection was more often with ultrasonic shears, the cystic duct was more often closed with ultrasonic energy and operation time was shorter (Table 1).

**Table 1:** Clinical and demographic characteristics for 1745 patients subjected to a laparoscopic cholecystectomy with a fundus first approach and a conventional technique.

	Fundus first n 1425	Conventional n 320	p
Age, mean years (SD)	46.8 (14.6)	46.4 (14.1)	0.771*
Female	1011 (70%)	211 (65%)	<0.001**
Emergent procedure	209 (14.7%)	21 (6.6%)	<0.001**
Laparoscopic operation converted into open surgery	30 (2.1%)	4 (1.2%)	0.462**
Daycare in elective surgery	1050 of 1216 (86.3%)	226 of 299 (75.6%)	<0.001**
Instrument for dissection:			<0.001**
Unipolar diathermy	7 (0.5%)	74 (23.5%)	
Ultrasonic shears	1401 (99.5%)	237 (76.5%)	
Ultrasonic shears closing the cystic duct	102 (7.2%)	10 (3.1%)	0.008**
Operating time, mean minutes (SD)	66.7 (33.4)	73.2 (28.1)	<0.001*

^*^Mann-Whitney U-test. **Chi-square test with Yates correction.

The 1745 LCs were performed by 29 surgeons. Individual surgeons operated between 1 and 492 LCs during the study period. Five surgeons performed more than 100 LCs each and together produced 1110 (64%) of the operations.

63 (3.6%) complications were registered: 10 (0.6%) bleedings, 33 (1.2%) infections and 12 (0.7%) bile leakages. Bile leakage was from the cystic duct in 8 patients and from an injured common bile duct in 4. The common bile duct injuries were partial, including less than 1/3 of the circumference. With the FF approach a common bile duct injury occurred in one of 1425 patients (0.07%) and with the conventional approach in 3 of 320 (0.9%) (p 0.003) (Table 2). Of these four patients three were female, and one was operated on due to an acute cholecystitis. In 230 emergent LCs one (0.4%) injury to the common bile duct occurred and in 3 (0.2%) of elective operations (p 0.484). Three injuries were treated by endoscopic placing of a stent in the common bile duct and in one an external drain was placed in the common bile duct. Four surgeons each had one common bile duct injury. Three of them operated between 184 and 492 LCs during the study period and one performed 61 operations.

**Table 2:** Common bile duct lesions in 4 (0.2%) of 1745 patients subjected to a laparoscopic cholecystectomy with a fundus first technique and a conventional technique.

	Fundus first n 1425	Conventional n 320
Common bile duct lesion	1 (0.07%)	3 (0.9%)

p 0.003, Chi-square test with Yates correction.

Leakage from the cystic duct was significantly more common when closure had been with ultrasonic shears than with any mechanical device such as clips or suture (Table 3).

**Table 3:** Bile leakage from the cystic duct in laparoscopic cholecystectomies, related to closure of the duct having been with ultrasonic shears or a mechanical device.

	Ultrasonic shears n 112	Mechanical closure n 1633
Cystic duct leakage	4 (3.6%)	4 (0.2%)

p < 0.001, Chi-square test with Yates correction.

A multivariate logistic regression model including conventional technique, patients age, female sex, year of surgery and emergent cholecystectomy as predictors of a common bile duct injury was created. Then a conventional technique starting dissection at the triangle of Calot was a significant risk factor for a common bile duct injury (Table 4).

**Table 4:** Results of a logistic regression model with common bile duct injury as outcome.

	B	S.E.	EXP (B)	95% CI for EXP (B)	p
Conventional technique	3.036	1.286	20.8	1.6–259.2	0.018
Age, years	0.068	0.040	1.1	0.99–1.15	0.086
Female	0.000	0.000	1.0	1,0–1.0	0.669
Year of surgery	1.135	1.287	3.1	0.25–38.7	0.378
Emergent cholecystectomy	3.036	1.286	20.8	0.25–38.8	0.378

**Figure 1:**
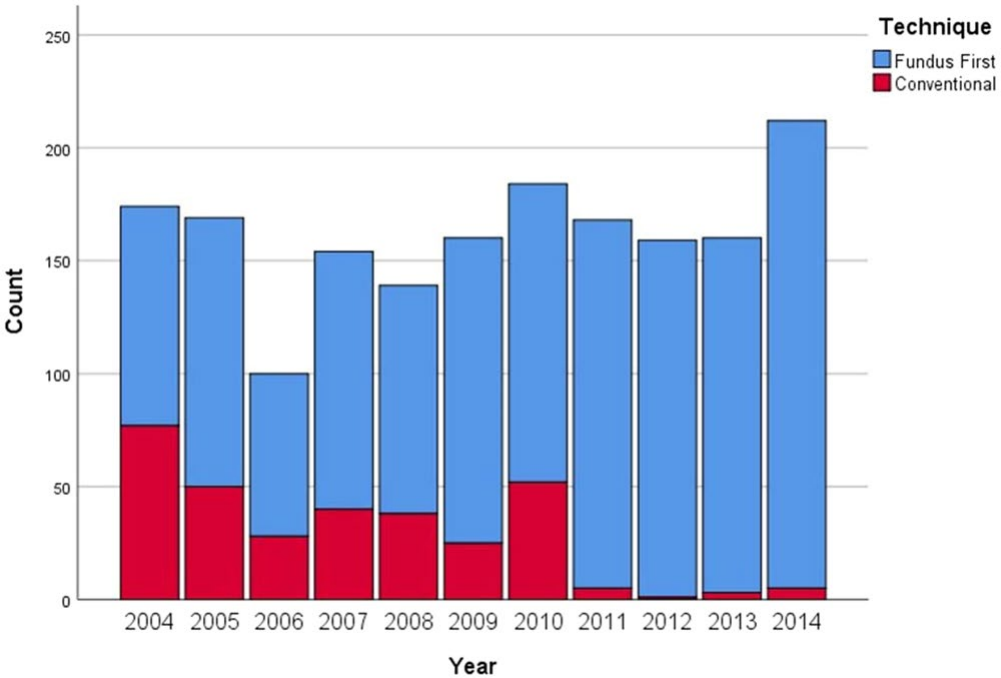
Laparoscopic cholecystectomies carried out by the Conventional vs Fundus first technique by year of study.

In the first year of the study period 56% of operations were with the FF technique and during the last four years 98% (Fig. 1).

## Discussion

The current series reflects the outcome of surgery for gallstone disease predominantly carried out in daycare. With the FF technique a higher proportion were completed in daycare than with the conventional technique. Also, the operating time was shorter. This is in congruence with previous reports on the effect of the FF technique^10,12,17,22,23^.

The predominant instrument for dissection with the FF technique was ultrasonic shears.

Concerning the postoperative complications, no significant difference between the FF technique and the conventional technique could be detected for the registered variables, except for common bile duct injury. With the FF approach a partial common bile duct injury occurred in 0.07% and with the conventional approach in 0.9%. In a logistic regression model, a conventional approach was an independent risk factor for common bile duct injury.

Although the study included 1745 cholecystectomies there were very few common bile duct injuries. However, the tenfold higher rate of bile duct injury with a conventional approach and the 20-fold increased risk in a multivariate model were statistically significant. It therefore seems relevant to conclude that the FF technique is safer than the conventional technique regarding a common bile duct injury. For all other registered complications, the methods produced similar results.

There was a higher proportion of emergent LC with the FF technique. Surgeons seem to have preferred the FF technique before the conventional technique in the emergent situation.

This is in congruence with an FF approach being preferred in potentially hazardous situations also in open surgery. In emergent LC the rate of bile duct injuries did not differ between the FF and the conventional techniques (Table 4).

Bile leakage from the sealed cystic duct occurred in 8 patients. If the cystic duct had been closed and divided with ultrasonic shears leakage was significantly more common than with a mechanical device closing the cystic duct. A series of patients were during the study period operated with a clipless technique, i.e. their cystic duct was divided and sealed with an ultrasonic device. This effort was based on a previous report^24^ indicating that regular clips would not be necessary. As it became evident that there was a high rate of bile leakage when the cystic duct had been closed with ultrasonic shears the method was abandoned. All patients developing a cystic duct leakage were successfully managed by transpapillary stent insertion.

Given the outcome of a previous trial^22^, dissection with ultrasonic shears was adopted as the preferred method for the dissection with the FF technique. This to some extent complicates the comparison between the study groups, where the imbalance in the choice between ultrasonic and electrosurgical energy is obvious. The electrosurgical device is widely used in LC, whereas the ultrasonic device has increasingly been used in wider and deeper operative fields. The potential risks and benefits related to ultrasonic dissection compared with the electrosurgical dissection for cholecystitis or cholecystolithiasis remains to be fully elucidated. However, in a recent meta-analysis comparing these major devices it was concluded that, the use of an ultrasonic device led to shorter operative time, less blood loss, fewer gallbladder perforations, shorter hospital stay and less abdominal pain/nausea. The numbers needed to treat to avoid one gallbladder perforation and postoperative nausea was 7 and 15, respectively^21^.

FF is often suggested as the procedure of choice for emergent operations and in situations where it is considered difficult or potentially hazardous to first address Calot’s triangle^9–16,18–20^. There are, however, reports that oppose the use of FF referring to a risk for complex, severe complications in the form of bile duct injuries combined with major vascular damage^25,26^. In these studies cholecystectomies began laparoscopically with no clear dissection plane existing between the liver bed and the gallbladder. The authors conclude that regarding the pathogenesis of these injuries, two prominent variables emerge: severe inflammation, and fundus-down (FF) cholecystectomy. The present results, based on a retrospective review of prospectively collected data from a decade of routine clinical practice, convey a completely different message. In fact, the FF technique was safer than the conventional surgical technique.

The FF technique was promptly adopted at the department and during the last four years only 2% of LCs were with a conventional technique. This probably reflects surgeons’ acceptance of the FF technique as an easy and safe method.

Laparoscopic surgery is technically demanding and requires specific psychomotor abilities and skills that differ from those in conventional surgery. All these skills may be difficult to obtain in the operating room. Novel teaching modalities and technologies have been introduced but the value of these compared with traditional patient‐based training remains to be endorsed^27–29^. Nevertheless, as part of the clinical research strategy to elucidate the value of FF, a project to comprehensively study the various components involved in the teaching and training of specialists and trainees to master the FF technique has been started (clinicaltrials.gov NCT03154164).

Further studies within this field are needed. The Swedish National Register for gallstone surgery (Gallriks) represents a robust way to document the outcome of FF for elective and emergent cholecystectomies and such studies are ongoing^30^. An additional aspect on the safety profile of FF in emergent cholecystectomy will be addressed in a recently launched multicenter RCT (clinicaltrials gov NCT03014817).

This is, to our knowledge, the largest series studied with the FF technique. Although representing a single center, the number of surgeons involved was quite high. Establishing the FF technique was effectively achieved and without hazardous complications or high conversion rates. With these convincing results it is of major interest to identify characteristics of the process and the author group currently discusses future studies on aspects of an effective process for introduction of a novel surgical techniques.

Although the registry documenting was prospectively done some data were retrieved retrospectively. Then there is a risk of a registration bias, the extent of which is difficult to estimate. However, this might be very limited since both the registry data and journal details were available and could be compared when necessary. Another factor probably minimizing the risk was that missing data could often be tracked.

Another potential shortcoming could be a reporting bias. The surgeon reported one technique used for the procedure and this is nearly impossible to question. In the normal case without a complication following this hardly represents a significant problem. However, since only one technique was registered this may cause a reporting bias as in a difficult dissection a combination of the techniques may have been used.

It is concluded that in laparoscopic cholecystectomy the fundus first technique, replacing a conventional technique, can be effectively introduced. The change into the fundus first technique is associated with lower rates of common bile duct injury. Clipless closure of the cystic duct increases the rate of leakage.

## Methods

All LCs prospectively registered in the Swedish National Register for gallstone surgery^31^ during 2004–2014 at a single surgical department (Sundsvall Hospital, Sundsvall, Sweden), were analyzed. Register data, if incomplete, were retrospectively completed from local patient files.

LC was performed with a standard technique using a 30° laparoscope through a 12-mm port just below the umbilicus, another 12-mm port beneath the xiphoid process, and two 5-mm working channels below the right subcostal arch. Pneumoperitoneum was created with an open technique, and the intraabdominal pressure was kept below 12 mmHg.

With the FF approach the visceral peritoneum was incised with ultrasonic shears from the infundibulum away from Calot’s triangle along the gallbladder bed up to the fundus; followed by dissection from the fundus down to the infundibulum. In this way, the gallbladder was left pedunculated by the cystic artery and duct. The cystic duct was most often divided between clips while the cystic artery was usually divided with the ultrasonic shears. The ultrasonic shears (Ultracision Harmonic ACE; Ethicon Endosurgery, Norderstedt, Germany) were set at level 3/5, and the optional effects maximum/minimum were according to the surgeon’s preference.

With the conventional technique a grasper placed in the most lateral port exerted traction on the gallbladder as the dissection started at the triangle of Calot, either with ultrasonic shears or with electrocautery. With the monopolar electrocautery, the blend cut or coagulation mode at 25 W was used according to the surgeon’s preference.

The critical view of safety with satisfactory exposure of the triangle of Calot was strived for irrespective of method used. All procedures included perioperative cholangiography. A retrieval bag was used for specimen extraction.

### Ethical approval, informed consent and Statistics

The study (No 2015/112-31) was approved by the Regional Research Ethics Committee, Umea° University, Umea°, Sweden. Thus, all methods were performed in accordance with the relevant guidelines and regulations.

As data was retrieved from The Swedish National Register for gallstone surgery (Gallriks), the Ethics Committee did not require informed consent for approval.

The SPSS (SPSS Inc, Chicago, IL, USA) software was used for statistical analysis. Mean and standard deviation were calculated. Fisher’s exact test and the Mann-Whitney U-test were used for the statistical analysis, as appropriate. A multivariate logistic regression model was created with appropriate registered variables as predictors of a common bile duct injury.

## Data Availability

The datasets generated during and/or analysed during current study are available from the corresponding author on reasonable request.
